# A novel homozygous variant in *MICOS13*/*QIL1* causes hepato‐encephalopathy with mitochondrial DNA depletion syndrome

**DOI:** 10.1002/mgg3.1427

**Published:** 2020-08-04

**Authors:** Yoshihito Kishita, Masaru Shimura, Masakazu Kohda, Masumi Akita, Atsuko Imai‐Okazaki, Yukiko Yatsuka, Yoko Nakajima, Tetsuya Ito, Akira Ohtake, Kei Murayama, Yasushi Okazaki

**Affiliations:** ^1^ Diagnostics and Therapeutics of Intractable Diseases Intractable Disease Research Center Juntendo University Graduate School of Medicine Tokyo Japan; ^2^ Department of Metabolism Chiba Children's Hospital Chiba Japan; ^3^ Division of Morphological Science Biomedical Research Center Saitama Medical University Saitama Japan; ^4^ Department of Pediatrics Fujita Health University School of Medicine Toyoake Japan; ^5^ Department of Pediatrics & Clinical Genomics Faculty of Medicine Saitama Medical University Saitama Japan; ^6^ Center for Intractable Diseases Saitama Medical University Hospital Saitama Japan

**Keywords:** cristae, MICOS complex, mitochondrial disease, mitochondrial DNA depletion syndrome

## Abstract

**Background:**

Mitochondrial DNA depletion syndrome (MTDPS) is part of a group of mitochondrial diseases characterized by a reduction in mitochondrial DNA copy number. Most MTDPS is caused by mutations in genes that disrupt deoxyribonucleotide metabolism.

**Methods:**

We performed the whole‐exome sequencing of a hepato‐encephalopathy patient with MTDPS and functional analyses to determine the clinical significance of the identified variant.

**Results:**

Here, whole‐exome sequencing of a patient presenting with hepato‐encephalopathy and MTDPS identified a novel homozygous frameshift variant, c.13_29del (p.Trp6Profs*71) in *MICOS13*. MICOS13 (also known as QIL1, MIC13, or C19orf70) is a component of the MICOS complex, which plays crucial roles in the maintenance of cristae junctions at the mitochondrial inner membrane. We found loss of MICOS13 protein and fewer cristae structures in the mitochondria of fibroblasts derived from the patient. Stable expression of a wild‐type *MICOS13* cDNA in the patients fibroblasts using a lentivirus system rescued mitochondrial respiratory chain complex deficiencies.

**Conclusion:**

Our findings suggest that the novel c.13_29del (p.Trp6Profs*71) *MICOS13* variant causes hepato‐encephalopathy with MTDPS. We propose that *MICOS13* is classified as the cause of MTDPS.

## Graphical abstract

We performed the whole‐exome sequencing of the hepato‐encephalopathy patient with MTDPS. The functional analyses revealed the clinical significance of the identified variant.

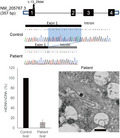

## 1 INTRODUCTION

Mitochondrial disorders affect approximately one in 5,000 live births and are linked to multiple organ failure. *MICOS13* encodes a protein that is one component of the mitochondrial contact site and cristae organizing system (MICOS) (van der Laan, 2012). Variants in *MICOS13* were recently reported to cause mitochondrial hepato‐encephalopathy (MIM: 616658) (Gödiker et al., 2018; Russell et al., 2019; Zeharia et al., 2016). All reported *MICOS13* indels or splicing variants led to frameshifts and the introduction of premature stop codons. Hepato‐encephalopathy patients commonly present with early onset fatal liver disease and encephalopathy. Hyperlactatemia and 3‐methylglutaconic aciduria are also common features of this disease. Some patients show nephrolithiasis or mild cardiac hypertrophy.

Variants in genes involved in mitochondrial DNA (mtDNA) replication (*POLG*, *TWNK*, *MGME1*, and *DNA2*), nucleic acid synthesis (*SLC25A4*, *TK2*, *SUCLA2*, *SUCLG1*, *RRM2B*, *DGUOK*, *MPV17*, *TYMP*, and *DTYMK*), and others (*AGK* and *FBXL4*) can cause MTDPS (Almannai, El‐Hattab, & Scaglia, 2018). Thus, genes responsible for MTDPS are largely limited to those involved in mtDNA replication and nucleic acid synthesis.

In the present study, whole‐exome analysis using next‐generation sequencing identified a novel *MICOS13* variant in a case exhibiting mitochondrial DNA depletion syndrome (MTDPS).

## 2 MATERIALS AND METHODS

### 2.1 Patient and informed consent

This study was approved by the regional Ethics Committees at Juntendo University, Saitama Medical University and Chiba Children's Hospital. We obtained written informed consent from the parents of the patient.

### 2.2 Genetic analysis

The whole‐exome sequencing (WES) was performed using a modified version of a previously described method (Kohda et al., 2016). Briefly, indexed genomic DNA (gDNA) libraries were prepared from gDNA of the patient's fibroblast cells, and exomes were captured using SureSelect V5 exome enrichment kits (Agilent Technologies), in accordance with the manufacturer's protocol. Sequencing was performed using 150‐bp paired‐end reads on a HiSeq2500 (Illumina). The quality of raw data was checked by FASTQC. After removing the low‐quality reads and adaptors, reads were mapped to the reference genome (GRCh37/hg19) with Burrows‐Wheeler Aligner (BWA), Picard, and SAMtools. GATK was also used for insertion and deletion realignment, quality recalibration, and variant calling. Detected variants were annotated using both ANNOVAR and custom Ruby scripts. Variants were filtered with minor allele frequencies (MAFs) of >0.5% for dbSNP, 1KG, the Exome Aggregation Consortium (ExAC), the Genome Aggregation Database (gnomAD), ESP6500siv2, and the 3.5KJPNv2 database from the Tohoku Medical Megabank Organization (ToMMo). PCR direct sequencing was performed using BigDye v3.1 cycle sequencing kit (Thermo Fisher Scientific) and Genetic Analyzer 3130xl (Thermo Fisher Scientific).

### 2.3 OXPHOS enzyme activity assays

Spectrophotometric enzyme activity assays using hepatic mitochondria were performed as previously described (Kirby, 2007). OXPHOS enzyme activities were measured using Cary 300 (Agilent Technologies) as per the manufacturer's instructions and were expressed as percentages of citrate synthase activity. Protein concentration was determined by the bicinchoninic acid assay (Pierce™ BCA Protein Assay Kit, Thermo Fisher Scientific).

### 2.4 Immunoblot analysis

Blue native (BN)‐PAGE, SDS‐PAGE, and western blot (WB) were performed as previously described (Kohda et al., 2016). The primary antibodies used were as follows: anti‐NDUFA9 (Complex I; #459100, Invitrogen), anti‐70 kDa Fp Subunit (Complex II; #459200, Invitrogen), anti‐core 1 (Complex III; #459140, Invitrogen), anti‐subunit 1 (Complex IV; #459600, Invitrogen), anti‐ATP Synthase beta (Complex V; A21351, Sigma‐Aldrich), anti‐V5 (R960‐25, Invitrogen), anti‐C19orf70 (MICOS13; SAB1102836, Sigma‐Aldrich), and anti‐C1orf151 (MICOS10; ARP44801, Aviva Systems Biology).

### 2.5 mRNA and mtDNA analyses

Quantitative reverse transcription PCR (qRT‐PCR) was performed for the analysis of mRNA. Primers were designed with Primer3 software. qRT‐PCR of cDNA extracted from human cells was performed using SYBR Premix Ex Taq (Takara), Power SYBR Green PCR Master Mix (Life Technologies), and Mx3000P (Agilent Technologies). The relative mRNA concentration was normalized to the average level of a housekeeping gene (GAPDH). mRNA expression was calculated from two experiments performed with more than three replicates.

Quantitative PCR of mtDNA was performed using primer sets for MT‐ND1 and CFTR (mtDNA gene MT‐ND1 was compared against a nuclear gene [CFTR exon 24]). A diagnosis of MTDPS was made when the relative copy number ratio of mtDNA to nuclear DNA was less than 35% of that in healthy control tissues in four independent experiments.

### 2.6 Electron microscopic observation

Control fibroblasts or fibroblasts from the patient were cultured on Aclar plastic film. The samples were fixated by immersion in 2.5% glutaraldehyde in 0.1 M phosphate buffer (pH 7.2) at 4°C for more than 1 h, and postfixated for 1 h in a solution of 1% osmium tetroxide in 0.1 M cacodylate buffer (pH 7.2). The samples were then dehydrated in graded ethanol and embedded in epoxy resin. Ultrathin sections were contrasted in uranyl acetate/lead citrate and the sections were observed under a JEM 1010 transmission electron microscope (JEOL).

### 2.7 OCR measurement

The oxygen consumption rate (OCR) was measured with the Seahorse XF96 extracellular flux analyzer (Agilent Technologies) as described previously (Shimura et al., 2019). Fibroblast cells were seeded in a 96‐well plate at 2 × 10^4^ cells/well with 80 μl of growth medium containing 25 mM glucose (Glu), and incubated for 24 h (37°C, 5% CO_2_). After replacing the medium with 160 μl of unbuffered DMEM containing 1 mM sodium pyruvate, 2 mM glutamine, and 25 mM glucose or 10 mM galactose (Gal), the assay plates were incubated at 37°C without CO_2_ for 1 h. Following the calibration of the sensor cartridge loaded with compounds including oligomycin (2 μM final concentration), carbonyl cyanide 4‐(trifluoromethoxy) phenylhydrazone (FCCP, 0.4 μM final concentration), and rotenone (1 μM final concentration), the experiments were started. The obtained data were normalized to the cell numbers determined using CyQUANT Cell Proliferation kit (Invitrogen). The data were calculated from two experiments performed with more than two replicates.

### 2.8 Rescue experiment

Cells were cultured at 37°C and 5% CO2 in Dulbecco's modified Eagle's medium (DMEM 4.5 g/L glucose or 1.0 g/L glucose; Nacalai Tesque) supplemented with 10% fetal bovine serum. Normal neonatal human dermal fibroblasts (Takara) and normal fetal human dermal fibroblasts (Toyobo) were used as control fibroblast cells. A rescue experiment was performed as previously described (Kohda et al., 2016). The open reading frame (ORF) of the *MICOS13* gene (NM_205767.3) was PCR amplified from cDNA. ORF and pTurboRFP‐mito (TurboRFP fused to a mitochondrial targeting sequence derived from subunit VIII of human cytochrome C oxidase; Evrogen) were cloned into the CS‐CA‐MCS lentiviral vector with a C‐terminal V5 tag, CAG promoter for mammalian cell expression, and blasticidin resistance using the In‐Fusion HD Cloning Kit (Clontech Laboratories, Inc.).

### 2.9 Statistics

Statistical differences were determined by two‐tailed Student's t‐test. A *p* value <0.05 was considered to be significant.

## 3 RESULTS

### 3.1 Case study

**Figure 1:**
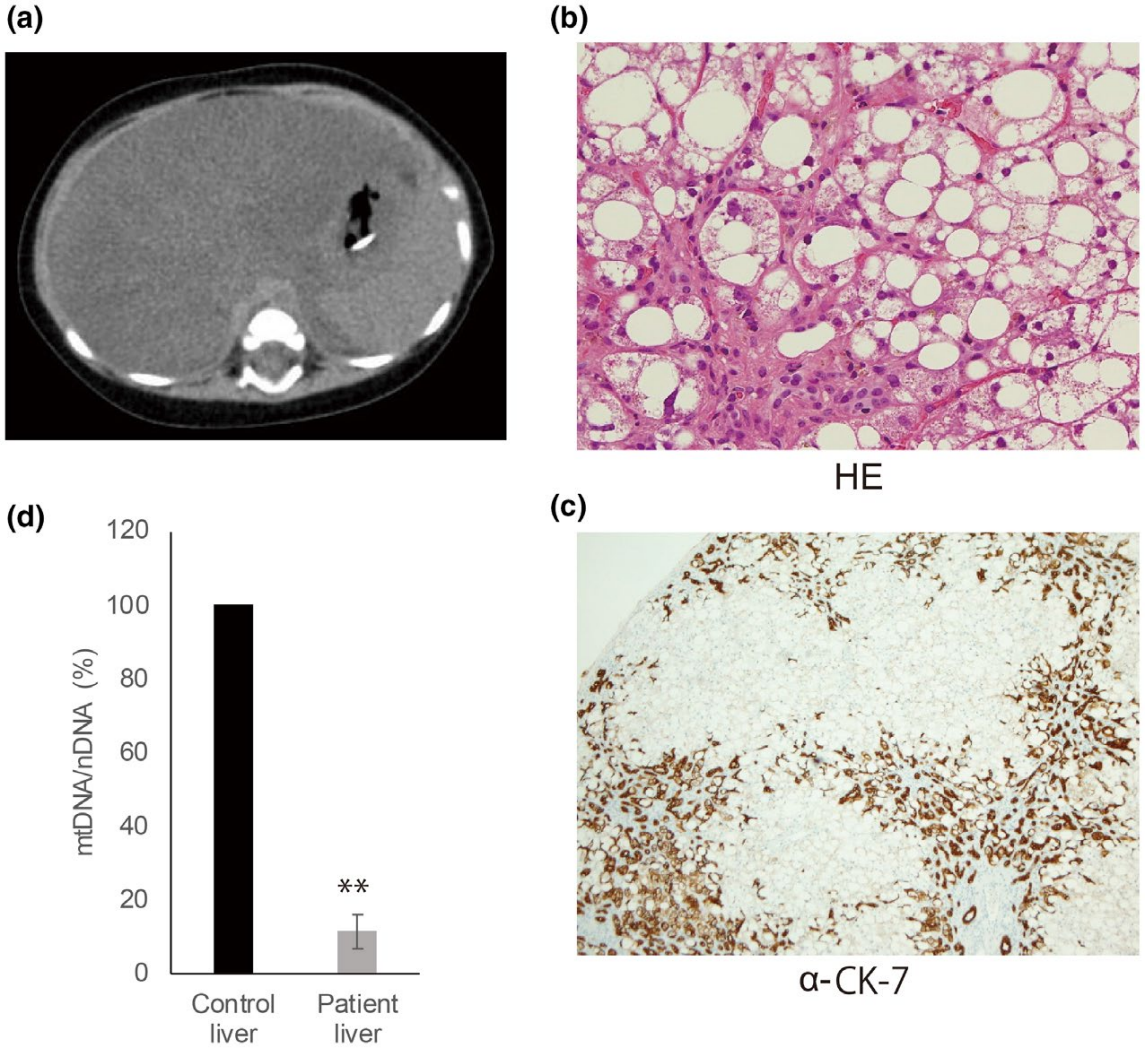
Clinical presentation of a patient with hepato‐encephalopathy and MTDPS. (a) Fatty liver was observed on abdominal CT. (b) Pathological observations by Hematoxylin and Eosin (HE) staining showed that large cytoplasmic fat vacuoles are diffusely distributed within hepatocytes. (c) Immunostaining with cytokeratin 7 (CK7) showed the proliferation of pseudobiliary ducts to be very prominent in the areas of fibrosis surrounding the liver lobules. The Glisson sheath showed centrilobular bile duct hyperplasia and the number of hepatic cells was small, indicating hepatic failure. (d) Hepatic mtDNA copy number was determined by qPCR. Quantitative PCR showed that liver mtDNA was remarkably decreased in the patient (mean ±SD: 11.5 ± 4.6%). Student's *t*‐test: **p < 0.01

A female infant (Patient ID: Pt94) was born to nonconsanguineous Japanese parents at 40 weeks and 3 days of gestation with a body weight of 2780 g (birth weight percentile 12.7). Breath‐holding was observed during crying from 3 months of age. At 4 months of age, abnormal liver function and hepatomegaly were observed, followed by poor feeding. The patient's liver was palpable at two finger‐breadths at 5 months of age. Lactic acid in the blood increased to 8 mM and decreased to 4 mM when milk was switched to medium‐chain triglyceride oil. Thrombocytopenia, worsened respiratory condition, and decreased coagulation function were observed, and she received treatment with albumin, fresh frozen plasma, and diuretics. Head computed tomography (CT) showed cerebellar atrophy. Head magnetic resonance imaging also showed mild atrophy of the cerebellum. Abdominal CT showed marked fatty liver and enlargement of the liver (Figure 1a). The patient's respiratory condition worsened and mechanical ventilation was required. Based on the abdominal CT imaging and an increase in serum Krebs von den Lungen 6, interstitial pneumonia was considered to have worsened. A neutrophil elastase inhibitor and an antimicrobial agent were administered for interstitial pneumonia, but without a clear beneficial effect. Cytomegalovirus antigen, β‐D glucan, and *Pneumocystis carinii* nucleic acid were negative, indicating no infection. Hypoproteinemia, increased ascites, and renal failure developed, and the patient died at 8 months old.

At 5 months of age, hypoproteinemia was evident with increased levels of hepatic enzymes, AST 489 and ALT 176, increased levels of bilirubin, triglyceride, and cholesterol; slightly increased levels of C‐reactive protein and ammonia; hypoglycemia and anemia; and decreased levels of platelets. Lactic acid and pyruvic acid levels were slightly increased, and the lactate/pyruvic acid ratio was 26.25 (lactate: 100 mg/dl = 11.1 mM pyruvate: 10 mg/dl = 1.14 mM) and the 3‐hydroxybutyric acid/acetoacetic acid ratio was 3.56. Amino acid analysis showed a decrease in the ratio between branched‐chain and aromatic amino acids (Fisher's ratio) and no other abnormal findings other than a slight increase in glutamic acid. Urine organic acid analysis showed a slight increase in the excretion of dicarboxylic acid, such as adipate, 3‐OH‐sebacate, and 3‐OH‐dodecanedioate. Tandem mass spectrometry was performed; however, there was no evidence of fatty acid abnormalities, and a liver disorder was strongly suspected. No abnormalities in chromosome G‐banding, urinary mevalonic acid, or very long‐chain fatty acids were observed. Gas chromatography/mass spectrometry analysis of organic acids in urine showed elevated excretion of 3‐methylglutaconic acid. We also found increases in the excretion of 4‐hydroxyphenyllactic‐2, fumarate, and malate.

Hepatic pathology showed marked fatty liver with bile duct proliferation (Figure 1b,c). There was a low number of normal hepatocytes. Most hepatocytes had diffuse large cytoplasmic vacuoles filled with lipids, indicative of hepatic failure. The pathology was consistent with a diagnosis of hepatic mitochondrial depletion syndrome.

Diagnostic analyses of liver tissue for mitochondrial respiratory chain (complexes I–IV) enzyme activities were performed. Only complex I was selectively decreased, and complexes III and IV, but not complex II, were considerably decreased (CI: 19.6%, CII: 59.8%, CIII: 30.2%, CIV: 30.4%). In addition, citrate synthase activity was more than double the normal level and the number of mitochondria was increased in compensation. The activity of each complex was expressed as the percentage of citrate synthase activity. A comparative quantitative analysis of mitochondrial and nuclear DNA showed that the ratio of mitochondrial DNA to nuclear DNA content was as low as 11.5% (Figure 1d). Therefore, the diagnosis of mitochondrial DNA depletion was made.

### 3.2 Identification of *MICOS13* variant

**Figure 2:**
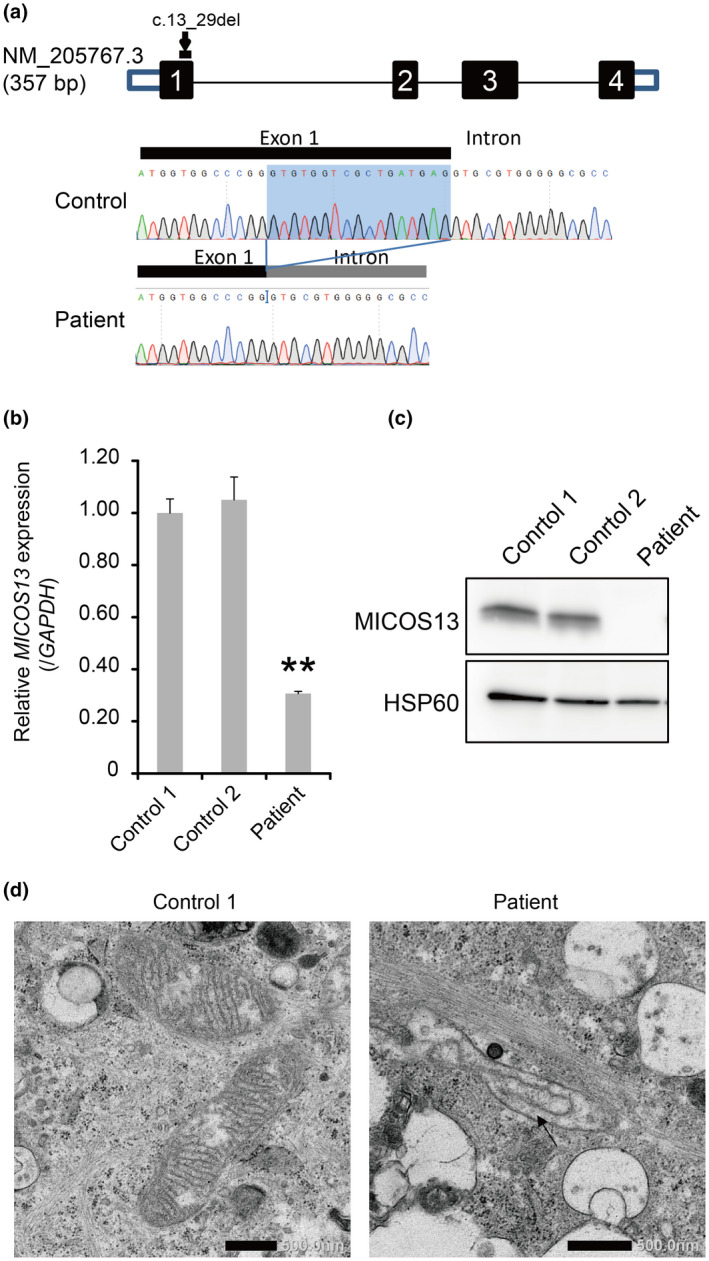
*MICOS13* mutation reduces *MICOS13* mRNA and protein levels and mitochondrial cristae organization in the MTDPS patient. (a) Location of the *MICOS13* mutation. The patient was homozygous for c.13_29del (p.Trp6Profs*71) in *MICOS13*. (b and c) Analysis of *MICOS13* mRNA and protein revealed that the mRNA was unstable and that MICOS13 protein was undetectable in the patient's fibroblasts. Student's *t*‐test: **p < 0.01. (d) Electron microscopy showed loss of cristae junction formation in the patient's fibroblasts. Scale bar = 500 nm

WES identified a homozygous variant, NM_205767.3: c.13_29del (p.Trp6Profs*71) in *MICOS13*, in the patient (Figure 2a). This 17‐bp deletion is located at the 3′ end of exon 1. The p.Trp6Profs*71 variant encodes a truncated, missense protein. The inheritance status could not be determined because parental samples were not available. Sanger sequencing confirmed the variant in the patient. Furthermore, SNP array (Kohda et al., 2016) and HDR (Imai et al., 2016; Imai‐Okazaki et al., 2017) analysis of WES data indicated long contiguous stretches of homozygosity (~4.3 Mb) around this variant (Figure S1).

### 3.3 Loss of MICOS13 and mitochondrial deficiencies

To investigate whether the variant has an effect on *MICOS13* expression, we analyzed the levels of *MICOS13* mRNA and protein. Quantification of *MICOS13* mRNA in the patient's fibroblasts by qRT‐PCR confirmed reduced levels (Figure 2b). SDS‐PAGE/western blot analysis of MICOS13 protein in the patient's fibroblasts showed loss of MICOS13 (Figure 2c). Transmission electron microscopy revealed loss of mitochondrial cristae structures in the patient's fibroblasts (Figure 2d).

### 3.4 Complementation assay

**Figure 3:**
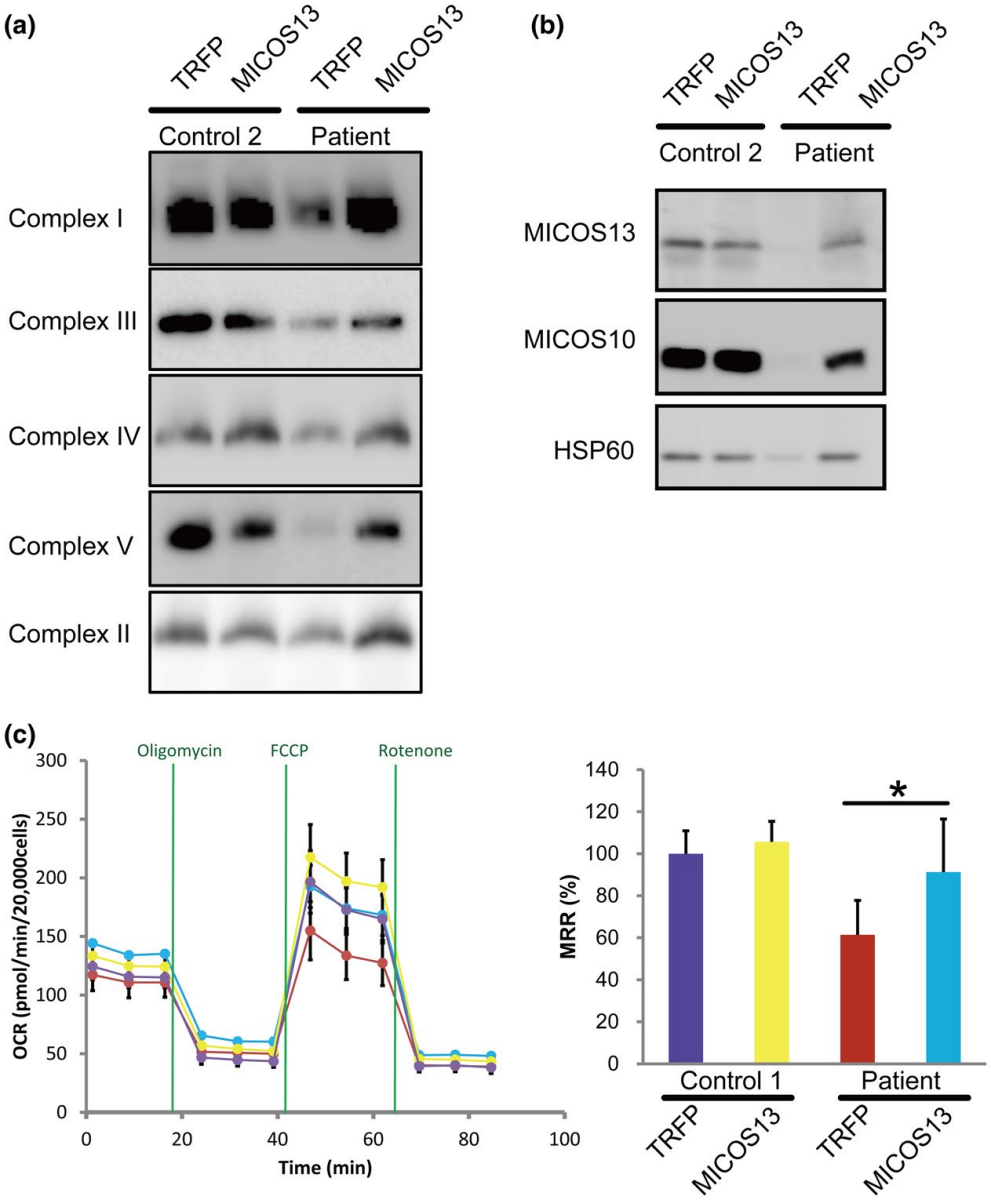
Expression of *MICOS13* in fibroblasts from the patient restored mitochondrial functions. (a and b) To confirm the causative role of MICOS13 loss in impaired mitochondrial function, we stably expressed a *MICOS13* cDNA using a lentivirus in fibroblasts from the patient. MICOS13 defects cause disrupted MICOS complex assembly and loss of MICOS10. MICOS13‐expressing cells showed increased levels of MICOS10. MICOS13 overexpression resulted in the restoration of complexes V and I formation. Complex II was used as a loading control for BN‐PAGE/WB. TRFP is a control vector expressing mitochondria‐targeted turboRFP. (c) MICOS13‐expressing cells displayed an increased oxygen consumption rate under galactose medium conditions. MMR: Maximum respiration rate. Student's *t*‐test: *p < 0.05

To further examine the effect of the variant, we stably expressed a wild‐type *MICOS13* cDNA in fibroblasts from the patient. We first analyzed the assembly of mitochondrial respiratory chain complexes in the patient's fibroblasts overexpressing MICOS13 by BN‐PAGE/western blotting. Overexpression of MICOS13 significantly rescued the assembly of mitochondrial complexes to levels comparable to those in control cells (Figure 3a). We also performed SDS‐PAGE/western blotting analysis of MICOS13 and MICOS10 (also known as C1orf151, MIC10, or MINOS1), because loss of MICOS13 results in MICOS disassembly with the degradation of MICOS10 (Guarani et al., 2015). Loss of MICOS13 and MICOS10 protein was observed in the patient's fibroblast, while the expression of MICOS13 in the patient's fibroblasts rescued this loss (Figure 3b). The OCR was significantly decreased in the patient's fibroblasts compared with that in control fibroblasts and this phenotype was rescued when the patient's fibroblasts were transfected with MICOS13 (Figure 3c).

## 4 DISCUSSION

In the present study, we found a new variant in *MICOS13* in a patient with liver failure, microcephaly, cerebellar atrophy, and pulmonary congestion associated with mitochondrial complex deficiencies and mtDNA depletion. Together with another case of hepatic mtDNA depletion (Russell et al., 2019), our report of *MICOS13* variation in MTDPS strongly indicates an association between mtDNA maintenance and MICOS13.

Our patient had hepatic disease and cerebellar atrophy (hepatic encephalopathy) as shown in other cases with *MICOS13* disease‐causing mutations (Gödiker et al., 2018; Russell et al., 2019; Zeharia et al., 2016). Organic acid analysis by gas chromatography/mass spectrometry also showed an increase in the urinary excretion of 3‐methylglutaconic acid, which was similar to that in previous cases. Mitochondrial respiratory chain complex enzyme activity assays showed a combined defect, indicating that defective MICOS13 affects multiple mitochondrial respiratory chain complexes. Notably, interstitial pneumonia was not reported in previous cases, although it is not clear whether MICOS13 was directly involved.

MICOS13 has not been reported to be directly involved in mitochondrial DNA replication; however, there are several reports to support this relationship. (a) IMMT (also known as MIC60 or Mitofilin) has a critical role in MICOS assembly and mitochondrial DNA organization (Yang et al., 2015). IMMT directly contacts mtDNA and is involved in the D‐loop architecture. (b) CHCHD10 is related to MICOS complex function and affects mitochondrial copy number. *CHCHD10* variants have been identified in cases with frontotemporal dementia‐amyotrophic lateral sclerosis (FTD‐ALS; MIM: 615903) (Genin et al., 2016). In human patient fibroblast cells with *CHCHD10* variants (Genin et al., 2016, 2018) and in a *Chchd10* knock‐in mouse (Genin et al., 2019), CHCHD10 defects resulted in decreased MICOS complex organization, reduced copy number, and instability of mtDNA. (c) ATAD3A is involved in mtDNA maintenance via the cholesterol metabolic pathway (Desai et al., 2017). ATAD3A interacts with the MICOS complex and MICOS complex formation is reduced in ATAD3A knockout mice (Peralta et al., 2018). This indicates that the maintenance of mtDNA is regulated through interaction between the MICOS complex and ATAD3A. Therefore, mtDNA depletion might be caused by MICOS complex disorganization due to MICOS13 loss. This was consistent with a diagnosis of mtDNA depletion syndrome for our case. Although it is important to fully understand the detailed molecular mechanisms of MICOS13‐related mtDNA replication, *MICOS13* should be classified as a causative gene of MTDPS.

## CONFLICT OF INTEREST

The authors declare no competing interests.

## AUTHOR CONTRIBUTIONS

MS, YN, TI, AO, and KM collected patient's data. YK, MK, and AIO analyzed the patient's genome data. YK, MS, MA, and YY performed the experiments. YK and YO designed the study. YK, MS, and YO wrote the manuscript.

## Supporting information

Fig S1

## References

[mgg31427-bib-0001] Almannai, M. , El‐Hattab, A. W. , & Scaglia, F. (2018). Mitochondrial DNA replication: Clinical syndromes. Essays in Biochemistry, 62(3), 297–308. 10.1042/EBC20170101 29950321

[mgg31427-bib-0002] Desai, R. Frazier, A. E. , Durigon, R. , Patel, H. , Jones, A. W. , Dalla Rosa, I. , … Spinazzola, A. (2017). ATAD3 gene cluster deletions cause cerebellar dysfunction associated with altered mitochondrial DNA and cholesterol metabolism. Brain, 140(6), 1595–1610. 10.1093/brain/awx094 28549128 PMC5445257

[mgg31427-bib-0003] Genin, E. C. , Bannwarth, S. , Lespinasse, F. , Ortega‐Vila, B. , Fragaki, K. , Itoh, K. , … Paquis‐Flucklinger, V. (2018). Loss of MICOS complex integrity and mitochondrial damage, but not TDP‐43 mitochondrial localisation, are likely associated with severity of CHCHD10‐related diseases. Neurobiology of Disease, 119, 159–171. 10.1016/j.nbd.2018.07.027 30092269 PMC7015038

[mgg31427-bib-0004] Genin, E. C. , Madji Hounoum, B. , Bannwarth, S. , Fragaki, K. , Lacas‐Gervais, S. , Mauri‐Crouzet, A. , … Paquis‐Flucklinger, V. (2019). ‘Mitochondrial defect in muscle precedes neuromuscular junction degeneration and motor neuron death in CHCHD10 S59L/+ mouse’. Acta Neuropathologica, 138(1), 123–145. 10.1007/s00401-019-01988-z 30874923

[mgg31427-bib-0005] Genin, E. C. , Plutino, M. , Bannwarth, S. , Villa, E. , Cisneros‐Barroso, E. , Roy, M. , … Augé, G. (2016). CHCHD 10 mutations promote loss of mitochondrial cristae junctions with impaired mitochondrial genome maintenance and inhibition of apoptosis. EMBO Molecular Medicine, 8(1), 58–72. 10.15252/emmm.201505496 26666268 PMC4718158

[mgg31427-bib-0006] Gödiker, J. , Grüneberg, M. , DuChesne, I. , Reunert, J. , Rust, S. , Westermann, C. , … Marquardt, T. (2018). QIL1‐dependent assembly of MICOS complex‐lethal mutation in C19ORF70 resulting in liver disease and severe neurological retardation. Journal of Human Genetics, 63(6), 707–716. 10.1038/s10038-018-0442-y 29618761

[mgg31427-bib-0007] Guarani, V. , McNeill, E. M. , Paulo, J. A. , Huttlin, E. L. , Fröhlich, F. , Gygi, S. P. , … Harper, J. W. (2015). QIL1 is a novel mitochondrial protein required for MICOS complex stability and cristae morphology. eLife, 4(MAY), 1–23. 10.7554/eLife.06265 PMC4439739 25997101

[mgg31427-bib-0008] Imai, A. , Kohda, M. , Nakaya, A. , Sakata, Y. , Murayama, K. , Ohtake, A. , … Ott, J. (2016). HDR: A statistical two‐step approach successfully identifies disease genes in autosomal recessive families. Journal of Human Genetics, 61(11), 959–963. 10.1038/jhg.2016.85 27357426 PMC5411490

[mgg31427-bib-0009] Imai‐Okazaki, A. , Kohda, M. , Kobayashi, K. , Hirata, T. , Sakata, Y. , Murayama, K. , … Ott, J. (2017). HDR‐del: A tool based on Hamming distance for prioritizing pathogenic chromosomal deletions in exome sequencing. Human Mutation, 38(12), 1796–1800. 10.1002/humu.23298 28722338

[mgg31427-bib-0010] Kirby, D. M. , Thorburn, D. T. , Turnbull, D. M. , & Taylor, R. W. (2007). Biochemical assays of respiratory chain complex activity In Mitochondria (2nd ed., pp. 93–119). Academic Press 10.1016/S0091-679X(06)80004-X 17445690

[mgg31427-bib-0011] Kohda, M. , Tokuzawa, Y. , Kishita, Y. , Nyuzuki, H. , Moriyama, Y. , Mizuno, Y. , … Okazaki, Y. (2016). A comprehensive genomic analysis reveals the genetic landscape of mitochondrial respiratory chain complex deficiencies. PLoS Genetics, 12(1), 1–31. 10.1371/journal.pgen.1005679 PMC4704781 26741492

[mgg31427-bib-0012] Peralta, S. , Goffart, S. , Williams, S. L. , Diaz, F. , Garcia, S. , Nissanka, N. , … Moraes, C. T. (2018). ATAD3 controls mitochondrial cristae structure in mouse muscle, influencing mtDNA replication and cholesterol levels. Journal of Cell Science, 131(13), 10.1242/jcs.217075 PMC6051345 29898916

[mgg31427-bib-0013] Russell, B. E. , Whaley, K. G. , Bove, K. E. , Labilloy, A. , Lombardo, R. C. , Hopkin, R. J. , … Bouchereau, J. (2019). Expanding and underscoring the hepato‐encephalopathic phenotype of QIL1/MIC13. Hepatology, 70(3), 1066–1070. 10.1002/hep.30627 30912852 PMC11108097

[mgg31427-bib-0014] Shimura, M. , Nozawa, N. , Ogawa‐Tominaga, M. , Fushimi, T. , Tajika, M. , Ichimoto, K. , … Murayama, K. (2019). Effects of 5‐aminolevulinic acid and sodium ferrous citrate on fibroblasts from individuals with mitochondrial diseases. Scientific Reports, 9(1), 1–11. 10.1038/s41598-019-46772-x 31332208 PMC6646320

[mgg31427-bib-0015] van der Laan, M. , Bohnert, M. , Wiedemann, N. , & Pfanner, N. (2012). Role of MINOS in mitochondrial membrane architecture and biogenesis. Trends in Cell Biology, 22(4), 185–192. 10.1016/j.tcb.2012.01.004 22386790

[mgg31427-bib-0016] Yang, R. F. , Sun, L.‐H. , Zhang, R. , Zhang, Y. , Luo, Y.‐X. , Zheng, W. , … Liu, D.‐P. (2015). Suppression of Mic60 compromises mitochondrial transcription and oxidative phosphorylation. Scientific Reports, 5, 1–8. 10.1038/srep07990 PMC4303897 25612828

[mgg31427-bib-0017] Zeharia, A. , Friedman, J. R. , Tobar, A. , Saada, A. , Konen, O. , Fellig, Y. , … Elpeleg, O. (2016). Mitochondrial hepato‐encephalopathy due to deficiency of QIL1/MIC13 (C19orf70), a MICOS complex subunit. European Journal of Human Genetics, 24(12), 1778–1782. 10.1038/ejhg.2016.83 27485409 PMC5117932

