# Supplementary figures and images for: A novel homozygous variant in MICOS13/QIL1 causes hepato‐encephalopathy with mitochondrial DNA depletion syndrome
Source: Mol Genet Genomic Med. 2020 Aug 4;8(10):e1427. doi: 10.1002/mgg3.1427 (PMC7549589; doi:10.1002/mgg3.1427)

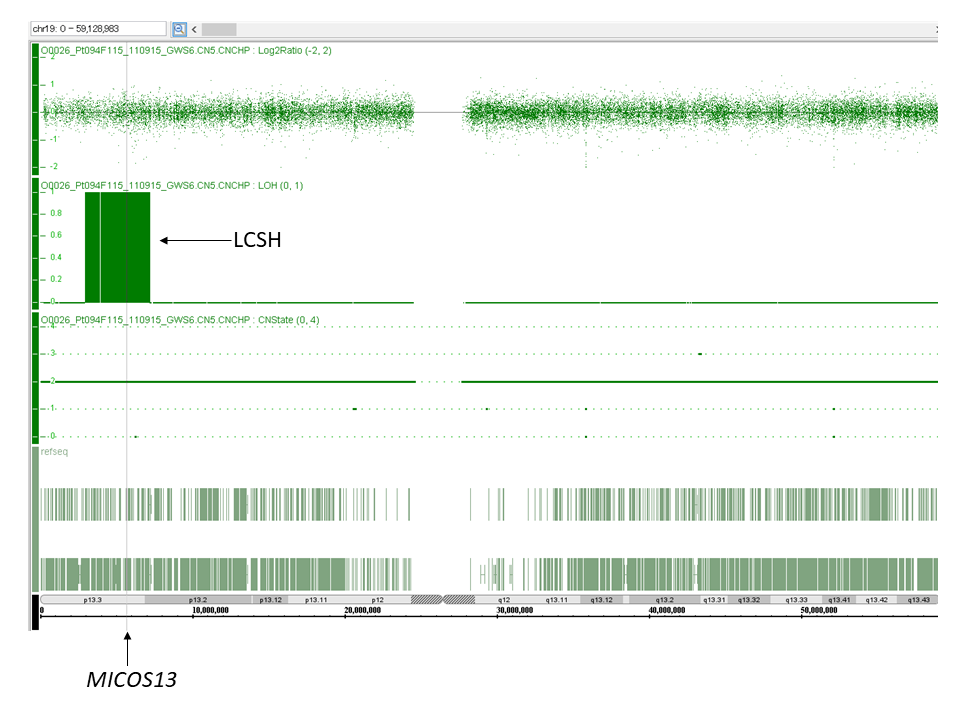

Supplement: Supplementary file 1 — Fig S1 [file MGG3-8-e1427-s001.png]
